# Characteristics and Outcomes of Patients in the Emergency Department with Left Ventricular Assist Devices

**DOI:** 10.5811/westjem.59733

**Published:** 2023-09-25

**Authors:** Alexander S. Finch, Michael M. Mohseni, Leslie V. Simon, Jennifer G. Finch, Lemuel E. Gordon-Hackshaw, Aaron B. Klassen, Aidan F. Mullan, David W. Barbara, Benjamin J. Sandefur

**Affiliations:** * Mayo Clinic, Department of Emergency Medicine, Rochester, Minnesota; † Mayo Clinic, Department of Emergency Medicine, Jacksonville, Florida; ‡ Mayo Clinic, Department of Nursing, Rochester, Minnesota; § University of Connecticut School of Medicine, Farmington, Connecticut; ∥ Mayo Clinic, Department of Quantitative Health Sciences, Rochester, Minnesota; ¶ Baycare Clinic, Department of Anesthesiology, Green Bay, Wisconsin

**Keywords:** emergency department, left ventricular assist device, outcomes, resuscitation

## Abstract

**Introduction:**

Left ventricular assist devices (LVAD) are increasingly common among patients with heart failure. The unique physiologic characteristics of patients with LVADs present a challenge to emergency clinicians making treatment and disposition decisions. Despite the increasing prevalence of LVADs, literature describing emergency department (ED) visits among this population is sparse. We aimed to describe clinical characteristics and outcomes among patients with LVADs seen in two quaternary-care EDs in a five-year period. Secondarily, we sought to evaluate mortality rates and ED return rates for bridge to transplant (BTT) and destination therapy (DT) patients.

**Methods:**

We conducted a retrospective cohort study of adult patients known to have an LVAD who were evaluated in two quaternary-care EDs from 2013–2017. Data were collected from the electronic health record and summarized with descriptive statistics. We assessed patient outcomes with mixed-effects logistic regression models including a random intercept to account for patients with multiple ED visits.

**Results:**

During the five-year study period, 290 ED visits among 107 patients met inclusion criteria. The median patient age was 61 years. The reason for LVAD implantation was BTT in 150 encounters (51.7%) and DT in 140 (48.3%). The most common presenting concerns were dyspnea (21.7%), bleeding (18.6%), and chest pain (11.4%). Visits directly related to the LVAD were infrequent (7.9%). Implantable cardioverter-defibrillator discharge was reported in 3.4% of visits. A majority of patients were dismissed home from the ED (53.8%), and 4.5% required intensive care unit admission. Among all patients, 37.9% returned to the ED within 30 days, with similar rates between DT and BTT patients (32.1 vs 43.3%; *P* = 0.055). The LVAD was replaced in three cases (1.0%) during hospitalization. No deaths occurred in the ED, and the mortality rate within 30 days was 2.1% among all patients.

**Conclusion:**

In this multicenter cohort study of ED visits among patients with an LVAD, dyspnea, bleeding, and chest pain were the most common presenting concerns. Visits directly related to the LVAD were uncommon. Approximately half of patients were dismissed home, although return ED visits were common.

Population Health Research Capsule What do we already know about this issue?

*Left ventricular assist devices (LVAD) are increasingly common, and therefore are more common among patients in the ED where clinicians face novel treatment concerns.*

What was the research question?

*We describe characteristics and clinical outcomes of patients with LVADs seen at included centers over a five-year period.*

What was the major finding of the study?

*There was a 37.9% return to ED rate in 30 days. Destination therapy and bridge to transplant return rates were 32.1% vs 43.3% (P = 0.055).*

How does this improve population health?

*Our study provides background on common chief concerns and outcomes, including rates of ED return, for patients with LVADs.*

## INTRODUCTION

With advancements in pharmacotherapy, mechanical devices, and surgical techniques, treatment options for advanced heart failure continue to expand. A left ventricular assist device (LVAD) is a continuous-flow device used in the setting of end-stage heart failure, with the goals of improving quality of life and longevity as destination therapy (DT) or as a cardiac bridge to transplant (BTT). From 2006–2016, a reported 22,866 LVADs were implanted internationally.^1^

Emergency physicians must be aware of the physiologic and anatomic changes inherent to patients with an LVAD and of the complications that may develop.^2^ ^–^ ^4^ Additionally, patients typically begin new heart and anticoagulant medications related to their device, which may result in adverse effects. Patients may seek evaluation in the emergency department (ED) for various LVAD-related concerns, as well as for concerns unrelated to the LVAD.^5^ Use of the ED by, and characteristics of, patients with an LVAD have been outlined in only three retrospective reports to our knowledge.^6^ ^–^ ^8^ Although these studies examined ED visits among LVAD patients, few guidelines and only one risk-stratification tool currently exist for identifying high-risk LVAD patients seeking emergency care.^9^

Bleeding, infection, thrombosis, and mechanical complications are among the many reasons for LVAD patients to seek care in the ED.^2^ ^,^ ^3^ ^,^ ^10^ ^,^ ^11^ Despite the increasing frequency of implantation of LVADs, relatively little is known regarding the proportion of ED visits that relate to these complications.^7^ Furthermore, given the scarcity of literature on this topic, it can be difficult for emergency clinicians to accurately diagnose and treat illness in an LVAD patient and subsequently ensure a safe disposition. Our primary aim in this study was to describe clinical characteristics and outcomes among a large cohort of LVAD patients seeking emergency care during a five-year period. A secondary aim was to compare mortality rates and risk of return to the ED within 30 days between BTT and DT patients.

## METHODS

### Study Design, Setting, and Participants

We conducted a retrospective cohort study of patients with an LVAD in place who were seen in two geographically distinct EDs of a single institution (Mayo Clinic Hospital-Saint Marys Campus in Rochester, Minnesota, and Mayo Clinic in Jacksonville, Florida) between January 1, 2013–December 31, 2017. All adult patients (≥18 years) who were registered as ED patients with implanted LVADs were eligible for inclusion in the study. The cohort size was determined by the number of encounters occurring during the study period, and each discrete ED visit was recorded. The two EDs are part of a multisite, quaternary-care academic institution with annual censuses of 74,000 and 30,000 during the study period. Our institutional review board approved the study protocol.

Patients were initially identified by searching our electronic health record (EHR) for patients who had *International Classification of Diseases, Ninth Revision* diagnosis code V43.21 (organ or tissue replaced by other means, heart assist device) on or before September 30, 2015, or *International Classification of Diseases, Tenth Revision, Clinical Modification* diagnosis code Z95.811 (presence of heart assist device) on or after October 1, 2015. To ensure that our search criteria identified all eligible patients, we cross-referenced these patients with an internal database of known LVAD patients at our hospital.^12^

We then reviewed data from the discrete ED encounters of patients with identified LVADs. Patients who did not have an LVAD implanted at the time of the ED encounter were excluded from the study. We also excluded patients with implanted LVADs who were directly admitted to the hospital and not evaluated in the ED. As a standard part of our admission process, all evaluated patients were asked for permission to use their documentation for research. Patients who declined research authorization were excluded from the study. We report our data in accordance with the STROBE (Strengthening the Reporting of Observational Studies in Epidemiology) guidelines for observational studies.^13^

### Data Sources and Management

Data were abstracted from the EHR by research team members. We defined all data fields a priori and developed a coding rubric. We used a standardized chart review process. Investigators responsible for abstraction of data (A.S.F., M.M.M., L.V.S., J.G.F., L.E.G.-H., and A.B.K.) were trained by the principal investigator (B.J.S.) and met at regular intervals to reconcile inconsistencies with the principal investigator. A sample of 15 visits was independently extracted by two investigators (M.M.M. and L.V.S.), and the interrater reliability was calculated for key variables and demonstrated with the Cohen κ statistic. The Cohen κ ranged from 0.8–1.0 for most variables and was within 0.6–1.0 for all variables, indicating good interrater reliability. We collected and managed study data by using the Research Electronic Data Capture tool hosted at our institution.^14^

### Variables and Outcomes

We reviewed all available data from each ED encounter, and we reviewed prehospital and referring hospital data when available. If a patient was admitted to the hospital from the ED, we reviewed the available inpatient record and dismissal summaries. Specific data that were collected included the following: 1) demographic information; 2) arrival method (emergency medical services vs private vehicle); 3) chief concern; 4) whether the encounter was specifically related to the LVAD; 5) whether the encounter was due to an LVAD-associated factor (eg, anticoagulant medication and bleeding); 6) antiplatelet medications; 7) anticoagulant medications; 8) implantable cardioverter-defibrillator discharge; 9) LVAD information including brand, model, placement date, and placement location; 10) indication for placement (BTT vs DT); 11) cardiac arrest or need for care in the ED; 12) disposition from the ED; 13) admission level of care; 14) duration of hospitalization; 15) 30-day repeat ED visits; 16) one-year repeat ED visits; 17) death within 30 days; and 18) in-hospital death.

Outcome measures were recorded up to one year after each discrete encounter. We categorized encounters as being related to the LVAD if they were specifically associated with a device complication (eg, device alarm, driveline injury, driveline infection).

### Statistical Methods

Continuous variables were summarized as mean (SD) or median (IQR); categorical variables were summarized as frequency (percentage). We performed comparisons of demographic characteristics between BTT and DT patients with two-sided Wilcoxon rank-sum tests for continuous features and χ^2^ tests for categorical features. We assessed ED visit outcomes with mixed-effects logistic regression models. Random intercepts were included to account for repeat visits to the ED by individual patients, and no random slopes were implemented.

Our main outcome of interest was return visit to the ED within 30 days after the primary visit; secondary outcomes were death in the ED and death within 30 days of patient discharge. For each of these outcomes, we fit independent univariable regression models using disposition (admitted vs dismissed) and therapy type (BTT vs DT) as the predictors of interest. Models were both unadjusted and adjusted for patient age, sex, and race. No variable selection or removal was performed. *P* < .05 was considered statistically significant. We conducted all analyses using R version 3.6.2 (R Foundation for Statistical Computing, Vienna, Austria).

## RESULTS

During the study period, 290 discrete ED encounters among 107 patients met our inclusion criteria. Patients were predominantly men (242; 83.4%), and the median age was 61 years (“IQR,” as in Methods 53–67 years). The median number of ED visits per patient was one, and the maximum was 17. Among included patients, 27 patients had one return visit (14 BTT, 13 DT), eight patients had two return visits (four BTT, four DT), three patients had three return visits (three BTT, zero DT), five patients had four return visits (two BTT, three DT), and four patients had five or more return visits (three BTT, one DT). Among discrete encounters, BTT (150 visits, 51.7%) and DT patients (140 visits, 48.3%) were similarly represented. The LVAD devices included HeartMate II (Abbott) (157 visits, 54.1%), HeartWare (Medtronic) (125 visits, 43.1%), and HeartMate 3 (Abbott) (seven visits, 2.4%), with one patient having no LVAD brand listed. Twenty visits (6.9%) were among patients whose LVAD was implanted at an institution other than the study sites.

The most common presenting concerns included dyspnea (21.7%), bleeding (18.6%), and chest pain (11.4%) (Table 1). Visits directly related to the LVAD were infrequent (23 visits, 7.9%). Implantable cardioverter-defibrillator discharges were noted in 19 visits (6.6%). The LVAD team was contacted by the ED team during 177 patient encounters (61.0%), although the LVAD team evaluated the patient in the ED in only 48 encounters (16.6%). Dismissal home from the ED was the most common disposition (53.8%). Only 13 encounters resulted in intensive care unit (ICU) admission (4.5%).

**Table 1.** Emergency department encounter characteristics.

Characteristic	No. of visits (%) (N = 290)
Presenting concern^a^	
Dyspnea	63 (21.7)
Bleeding	54 (18.6)
Epistaxis	25 (8.6)
Hematemesis	1 (0.3)
Hematochezia	13 (4.5)
Melena	25 (8.6)
Other	12 (4.1)
Chest pain	33 (11.4)
Syncope	21 (7.2)
ICD discharged	19 (6.6)
Fall	13 (4.5)
Fever	12 (4.1)
Weakness	9 (3.1)
Leg pain	9 (3.1)
PICC problem	7 (2.4)
LVAD alarm	5 (1.7)
Cough	4 (1.4)
Headache	4 (1.4)
Abdominal pain	4 (1.4)
Altered mental status	3 (1.0)
Rash	3 (1.0)
Back pain	3 (1.0)
Arm pain	3 (1.0)
Stroke/stroke symptoms	3 (1.0)
NPWT not working	2 (0.7)
Other concern (1 occurrence each)	28 (9.7)
Unknown	0 (0)
LVAD directly related to visit	
Yes	23 (7.9)
No	265 (91.4)
Unknown	2 (0.7)
LVAD team involvement	
LVAD team contacted	
Yes	177 (61.0)
No	111 (38.3)
Unknown	2 (0.7)
LVAD team evaluation in ED	
Yes	48 (16.6)
No	220 (75.9)
Unknown	22 (7.6)
Disposition	
External facility	0 (0)
Dismissed home	156 (53.8)
Hospital admission	109 (37.6)
Hospital observation	12 (4.1)
ICU admission	13 (4.5)
Unknown	0 (0)

*ED*, emergency department; *ICD*, implantable cardioverter-defibrillator; *ICU*, intensive care unit; *LVAD*, left ventricular assist device; *NPWT*, negative pressure wound therapy (wound vac); *PICC*, peripherally inserted central catheter.

^a^ Patients could have >1 concern per visit.

Among all patients admitted to the hospital or ICU (122), the median duration of hospitalization was one day. During hospitalization, the LVAD rarely required replacement (two cases, 1.6%; 95% confidence interval [CI] 0.3–6.5%). Among all ED encounters, 110 (37.9%) (95% CI 32.4–43.8%) resulted in return to the ED within 30 days. Among the 156 patient encounters that resulted in dismissal from the ED, 68 (43.6%; 95% CI 35.7–51.8%) returned to the ED within 30 days. After adjusting for patient age, sex, and race, patients dismissed from the ED were nearly twice as likely to return to the ED within 30 days (odds ratio [OR], 1.81; 95% CI 1.01–3.27; *P* = 0.047) than were those admitted to the hospital or ICU (Table 2). Age, sex, and race were not significant predictors of ED return.

**Table 2.** Multivariable analysis of overall 30-day ED returns.

Characteristic	Odds ratio (95% CI)	*P*-value
Age, per 1-year increase	1.00 (0.97–1.04)	0.84
Sex		0.95
Women	Reference	
Men	1.03 (0.40–2.66)	
Race		0.49
Other than White	Reference	
White	0.77 (0.36–1.63)	
ED disposition		.05
ICU or hospital admission	Reference	
Dismissal or hospital observation	1.81 (1.01–3.27)	

*ED*, emergency department; *ICU*, intensive care unit.

Among all patients, no deaths occurred in the ED. The overall 30-day mortality rate for the cohort was six patients (2.1%). No significant difference in 30-day mortality rate was found between the BTT (three, 2.0%) and DT (three, 2.1%) groups after accounting for repeat visits (*P* = 0.92).

The DT patients were significantly older than the BTT patients, with mean ages of 65.4 years and 55.3 years, respectively (*P* < .001) (Table 3). In a univariable analysis, DT patients were 37.4% less likely than BTT patients to return to the ED within 30 days, although the comparison did not reach significance (OR, 0.63; 95% CI 0.39–1.01; *P* = 0.056). Similarly, when accounting for repeat visits to the ED by discrete patients (OR, 0.59; 95% CI 0.29–1.20; *P* = 0.15) and after accounting for patient age, sex, and race, (OR, 0.51; 95% CI 0.23–1.14; *P* = 0.10), no differences in 30-day return visits to the ED were observed between the BTT and DT groups.

**Table 3.** Univariable analysis of emergency department encounters by therapy type.

	Therapy type^a^		
Characteristic	Bridge to transplant (n = 150)	Destination (n = 140)	*P*-value
Age, years	55.3 (9.9)	65.4 (10.3)	<.001
Sex			
Men	117 (78.0)	125 (89.3)	0.01
Women	33 (22.0)	15 (10.7)	
Race			<.001
Black	50 (33.3)	7 (5.0)	
White	86 (57.3)	116 (82.9)	
Other	11 (7.3)	2 (1.4)	
Unknown	3 (2.0)	15 (10.7)	
Visit outcome			
30-day ED return	65 (43.3)	45 (32.1)	0.06
1-year ED return	122 (83.0)	103 (73.6)	0.13
30-day death	3 (2.0)	3 (2.1)	0.92

*ED*, emergency department.

^a^ Values are mean (SD) or No. of visits (%).

## DISCUSSION

Our study describes characteristics of patients with LVADs seen in the EDs of two large quaternary-care centers of the same institution. The BTT and DT patients were evenly represented in our cohort. Unlike in previous studies, which have included only locally implanted devices, 6.9% of patients in our study cohort had LVADs implanted at institutions other than the study sites.^6^ ^,^ ^7^ To our knowledge, we are the first to report return rates among LVAD patients dismissed from the ED: 43.6% within 30 days of the index visit. When patients were admitted, the median duration of admission was brief (one day); however, dismissal from the ED nearly doubled the risk of a return visit to the ED within 30 days. Similar to findings of other investigations,^7^ no deaths in the ED were observed.

Regarding disposition from the ED, the proportion of the population dismissed home from the ED (53.8%) was higher than that reported in a previous study (13.4%).^7^ This may represent practice site variation or an evolution in the current standard of care for LVAD patients seeking immediate care for acute concerns. However, the high risk of 30-day ED return observed in this cohort, among all encounters (37.9%) and among ED encounters resulting in dismissal (43.6%), suggests that clinicians should be aware of the high likelihood of an ED return visit within 30 days.^15^

Our report is novel in its characterization of ED encounters by BTT and DT groups. Prior studies on this topic specific to the ED have examined LVAD patients only in aggregate.^6^ ^,^ ^7^ Our study expands on previous work, including a recent study of more than 44,000 ED visits in which investigators sought to derive and validate a novel prediction score for death by separating patients into these key subgroups.^9^ A patient-centric approach including the intention of device implantation is beneficial in the clinical approach to LVAD patients, and this may be especially useful when characterizing long-term outcomes.^1^ ^,^ ^16^

No difference in mortality rate was observed between the BTT and DT groups. We found a 30-day mortality rate of 2.1% in the study group, which was lower than that in comparable studies on the topic.^1^ ^,^ ^16^ In comparison, Piffard and colleagues^17^ found a 22.9% mortality rate in ICU patients after LVAD implantation, although our study focused on patients admitted through the ED. As can be inferred from these data, LVAD patients are at substantial risk for worsening of clinical status after being hospitalized. We found that ICU admission was uncommon (13, 4.5%), and we identified no predictive factors for ICU admission in our cohort. A clinical implication of our study is that these patients may be safer for dismissal than previously thought, although they remain at risk for death within 30 days. Finally, DT patients were 37.4% less likely than BTT patients to return to the ED within 30 days. Unfortunately, our data do not provide an explanation for this observation, which is an area for potential future study.

In our study, ED encounters were more likely to result from LVAD-associated concerns such as bleeding in the setting of anticoagulation therapy (54, 18.6%) than from concerns directly related to LVAD function, which were uncommon (23, 7.9%). Our findings are consistent with those of other reports on this topic. One study showed bleeding to be the most common presenting concern in their analysis of 620 ED encounters with LVAD patients: 182 visits for bleeding (29.4%) were noted, compared with only 52 device-specific visits (8.4%).^7^ Tainter and colleagues^6^ similarly found device alarm or malfunction to account for only 4% of visits, whereas chest pain, syncope, and bleeding were common among their patient cohort.

## LIMITATIONS

We note important limitations to our study. The retrospective nature of the investigation at our two study sites limited our ability to obtain data, which could only be obtained from the existing EHR. The research team was trained by the principal investigator to minimize variation in data input, as evidenced by high interrater reliability calculations. Although we attempted to broaden the generalizability of our findings by including two geographically distant sites, all patients were cared for within the same hospital system. Increasing the number and diversity of participating sites would improve future investigations. However, this study is the first, to our knowledge, to include patients with LVADs implanted at institutions other than the study sites, which potentially improves its generalizability compared with the existing literature. Nevertheless, we were unable to determine time from implantation to ED visit, and so we could not assess whether this was an important factor.

A notable feature of our data set is the inclusion of patients with the HeartMate 3 device, which has not been previously reported in other ED-based studies. Additionally, the HeartWare device was better represented in our study than in earlier investigations.^6^ ^,^ ^7^ Furthermore, although BTT and DT encompass most indications for LVAD placement, we did not identify any patients in our cohort with an LVAD implanted as a bridge to recovery. Because our study included patients with LVADs placed at other facilities, this potentially may have affected our admission and outcome data. Additionally, our sites may have a level of expertise with LVADs in general that may not be generalizable to other settings.

Regarding our statistical methods, we did not detect a significant difference between our groups of interest, although our study may have been underpowered to detect a small and true difference in the groups. Although we did evaluate return visits, we could not discern whether a patient visited an outside ED during the subsequent 30 days after the index visit. This is a limitation of our study design, but any additional ED visits that occurred outside of our institutions would only serve to reinforce our findings. Finally, patients were closely followed up by the LVAD coordinator team; therefore, it is unlikely that any patient death would have been unnoticed. It is possible, however, that a patient death could have been missed, particularly for those with devices placed at an outside hospital.

## CONCLUSION

Among LVAD patients seen in two quaternary-care EDs of one institution, most visits were for LVAD-associated concerns such as bleeding, as opposed to visits directly related to the device itself. More than half of the cohort was dismissed home, although LVAD patients cared for in the ED had a high rate of return regardless of disposition. Among those who were dismissed, we found a 43.6% rate of return to the ED within 30 days.
